# Depression in geriatrics: a systematic review and meta-analysis of prevalence and risk factors in Egypt

**DOI:** 10.1186/s12877-024-05484-2

**Published:** 2024-11-11

**Authors:** Hebatalla Ahmed, Heba Khaled, Ahmed A. Allam, Bassima Alodini, Ahmed Azzam, Anees Adel Hjazeen, Hassan Samy Hassan, Sarah Mohamed Hussein, Fatma E. Hassan

**Affiliations:** 1 Department of Public Health, Faculty of Medicine, Kafrelshiekh University, Kafrelshiekh, Egypt; 2 Department of Biochemistry, Faculty of Pharmacy, Cairo University, Cairo, Egypt; 3 Department of Biology, Biotechnology Program, the American University in Cairo, New Cairo, Egypt; 4 Biomedicine, Uppsala University, Uppsala, Sweden; 5 Department of Microbiology and Immunology, Faculty of Pharmacy, Helwan University, Cairo, Egypt; 6 Biostatistician at Royal Medical Services, Amman, Jordan; 7 Faculty of Pharmacy, Tanta University, Tanta, Egypt; 8 Department of Public Health, Community Medicine, Environmental and Occupational Medicine, Faculty of Medicine, Suez Canal University, Ismailia, Egypt; 9 Medical Physiology Department, Faculty of Medicine, Kasr Alainy, Cairo University, Giza, 11562 Egypt; 10 General Medicine Practice Program, Department of Physiology, Batterjee Medical College, Jeddah, 21442 Saudi Arabia

**Keywords:** Depression, Egypt, Geriatrics, Meta-analysis, Older adults, Prevalence, Risk factors, Systematic review

## Abstract

**Background:**

Depression is the most common psychiatric disorder in older adults, even though it is commonly misdiagnosed and undertreated, leading to exacerbations of preexisting medical conditions and even a higher mortality rate. In the present systematic review with meta-analysis, we quantify the magnitude of depression and its associated risk factors among the older adult population in Egypt.

**Methods:**

A thorough literature search was performed from 2010 up to October 2023. The results were presented as proportions or risk difference with a 95% confidence interval (CI) calculated using the random effects model. A sensitivity analysis was performed to examine the robustness of the results.

**Results:**

Our study included 14 articles with 5857 older adults published between 2011 and 2023. All the included studies assessed depression in geriatrics using the Geriatric Depression Scale. Of the 14 studies, 5 were for community-dwelling older adults, 3 were for older adults attending primary health care (PHC) centers, 2 were for hospitalized older adults, 3 were for residing in geriatric homes, and one for residing in geriatric homes, hospitalized older adults, and community-dwelling older adults. The overall prevalence of depression among Egyptian geriatrics was 64.6%. The pooled prevalence of depression was 59.6%, 67.0%, 67.0%, and 62.0% for community-dwelling older adults, older adults people attending PHC, hospitalized older adults, and older adults residing in geriatric homes, respectively. Older adults with chronic illness, female sex, and low-income elders and elders who were not employed had a higher risk for depression with pooled risk differences of 34.9%, 17.8%, 23.8%, and 15.1% (*P* < 0.05), respectively. In contrast, there was no significant difference in risk for depression in the older adults residing in urban areas compared to rural areas, the older adults aged + 70 compared to those less than 70, individuals with low levels of education or who are illiterate compared with those with higher levels of education and the older adults who live alone compared with those living with family.

**Conclusion:**

More than half of the older adults in Egypt suffer from depressive symptoms. Chronic diseases, female sex, unemployment, and low-income inequality are the most significant factors contributing to depression among Egypt’s older adults.

**Supplementary Information:**

The online version contains supplementary material available at 10.1186/s12877-024-05484-2.

## Background

Globally, the total number of individuals with depression is estimated to exceed 280 million [1]. The World Health Organization (WHO) states that depression was the leading cause of global disability in 2015, accounting for 7.5% of all years lived with a disability [2]. Additionally, depression plays a significant role in contributing to the staggering number of over 800,000 suicides that take place annually [2].

Ageing does not necessarily bring on depression. However, older adults persons are more likely to suffer from depression [3]. It is influenced by various risk factors encompassing psychological, physical, and social aspects. Female sex, marital discordance, low socioeconomic class, the presence of chronic illnesses, life stressors, low education levels, and social withdrawal are among the frequent risk factors [4].

The global prevalence of depression among older adults exhibits significant variation, with a higher prevalence observed in developing countries compared to their developed counterparts [5]. According to national-level meta-analyses, the prevalence estimations of depression among the older populations in India, Iran, and China were found to be 34.4%, 43.0%, and 23.6%, respectively [6–8]. However, no quantifiable data on depression and its associated risk factors among Egyptian geriatrics is available.

Depression among older adults in Egypt is shaped by a complex interplay of healthcare, social, and economic factors. The country’s under-resourced mental health system limits access to specialized care, delaying early detection and treatment of depression. Additionally, rapid social changes, such as increasing urbanization and the shift from extended to nuclear family structures [9], have weakened traditional support systems that once offered protection against social isolation. Moreover, the ageing population in Egypt is expected to double by 2050 [10], placing additional strain on an already limited healthcare infrastructure. Economic challenges further exacerbate the issue, as many older adults face financial insecurity due to limited incomes and rising healthcare costs.

Compounding these challenges is the pervasive cultural stigma surrounding mental health in Egypt [11]. Deeply rooted in cultural, religious, and societal norms, this stigma discourages many older adults from seeking help for mental health issues. Given the unique social and cultural context of Egypt, we conducted this meta-analysis to provide a comprehensive understanding of the prevalence and risk factors of depression among older adults in Egypt. By focusing specifically on Egypt, this study addresses a gap in global research and offers insights to help policymakers prioritize mental health issues and guide targeted interventions for this vulnerable population.

## Methods

### Search strategy

A thorough literature search was conducted for studies published between 2010 and August 2023 using the following databases: MEDLINE [PubMed], Scopus, Google Scholar, and Web of Science. The search utilized the following keywords: (“Depression” OR “depressive disorder” OR “mood disorder”) AND (“geriatrics” OR “older adults” OR “ageing” OR “older adults” OR “seniors”) AND (Egypt). Additionally, the reference lists of the included studies were scanned to ensure a thorough representation of the existing literature. Table S1 shows the 27 items of the PRISMA checklist. The time frame of 2010–2023 was selected to analyze depression trends among older adults in Egypt using the most recently available and robust data. Before 2010, there was limited research published on geriatric mental health issues in the country. Studies from that era tended to have methodological limitations.

## Eligibility criteria

### Inclusion criteria

#### The study was included if it met the following criteria

(1) Involving the older adult Egyptian population aged 60 years and older. (2) Reporting the prevalence of depression in older adults. (3) Using a rigorously validated screening tool with a well-defined and established cut-off point for identifying depressive symptoms, these tools have been thoroughly evaluated for their psychometric properties to ensure reliability and validity in accurately assessing depressive symptoms in older adult populations.

### Exclusion criteria

Studies that have not reported the screening tool or those with unclear cut-off points of the screening tool were excluded. Two independent reviewers screened eligible articles [A.A.] and [H.K.] from the electronic search outputs based on the aforementioned inclusion and exclusion criteria. Disagreements were solved by discussion and consensus between the two reviewers.

### Data extraction

In the data collection section, [F.E.H.] and [S.M.H.] performed the initial data extraction, while a third reviewer, [H.A.], conducted a cross-check to ensure consistency and accuracy. From each study included in the analysis, the following key details were carefully documented: last name of the first author, publication time, region, study design and setting, assessment tool, totally examined sample size, prevalence (%), and risk factors associated with depression. Additional items that were crucial for conducting a meta-analysis on risk differences were extracted.

### Quality assessment

We adopted JBI’s critical appraisal tools for prevalence studies to assess the quality of the included studies [10]. The checklist items are presented in Table S2.

### Data synthesis

The results were presented as proportions or risk differences with a 95% confidence interval (CI) calculated using the random effects model. Heterogeneity between the studies was assessed using I-squared and Cochran’s Q statistics. To examine the robustness of the results, sensitivity analysis was performed utilizing the leave-one-out technique. Publication bias was not assessed as it does not produce reliable outcomes for meta-analysis of the proportion [12]. All statistical analyses were performed using Comprehensive Meta-Analysis version 3.0 (Biostat, Englewood, NJ, USA).

## Results

### Study selection and characteristics of included studies

**Fig. 1:**
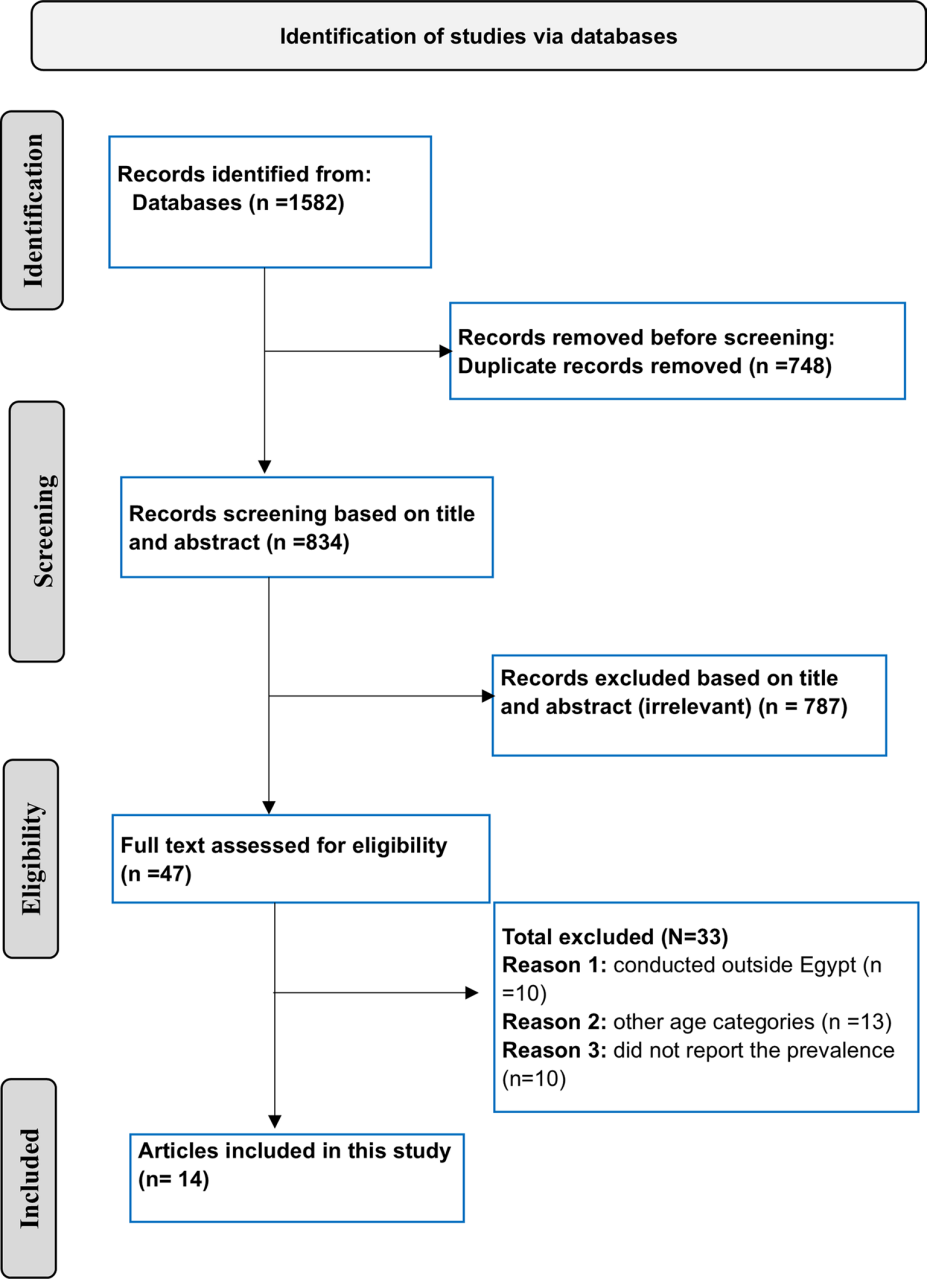
Flow chart depicting the selection of publications

A total of 1582 titles were identified through database searches, of which 14 were included in this review (Fig. 1) [13–26]. The current systematic review comprises 14 studies with 5857 older adult individuals. Thirteen of the included studies assessed depression in geriatrics using the shorter version of the Geriatric Depression Scale (GDS-15), with a cut-off score of more than 4 suggesting depression. Only one study used the longer version of the Geriatric Depression Scale (GDS-30), with a cutoff score of more than 9, suggesting depression [26]. Of the 14 studies: 5 were for community-dwelling older adults [15, 16, 21, 22, 24], 3 were for older adults people attending PHC centers [14, 19, 26], 2 for hospitalized older adults [20, 25], and 3 for older adults residing in geriatric homes [13, 17, 23]. As well there was one study for older adults residing in geriatric homes, hospitalized older adults, and community-dwelling older adults [18]. Table 1 shows the characteristics of the included studies. The overall quality of the included studies was quite fair, as presented in Table S3.

**Table 1:** Characteristics of the included studies

Author	Publication time	Region	Study design	Study setting	Assessment tool Cut-off	Total	Prevalence (%)	Risk factors
Zorkany [16]	2020	Gharbia	CS	Community (rural)	GDS-15 > 4	200	88.5	Smoking habits, low income, absence of persons, having diseases, older age, sleep habitat, loneliness, and lack of any help.
Gilany [15]	2018	Mansoura	CS	Community (urban and rural)	GDS-15 > 4	487	43.7	Urban residence, insomnia, being a woman, low income, disturbed marital life, dependent old adults, and absence of religiosity.
Abdo [24]	2011	Zagazig	CS	Community (urban and rural)	GDS-15 > 4	290	46.6	Increased age, being female, not married, having previous death events among the surrounding, age group over 75 years, and low socioeconomic condition.
Aly [22]	2018	Sohag	CS	Community (urban and rural)	GDS-15 > 4	1027	62.7	Increased age, female sex, and living in rural areas.
El-Sherbiny [21]	2016	Fayoum	CS	Community (urban and rural)	GDS-15 > 4	2219	74.5	Female sex, with increased age, and disease burden.
Ahmed [17]	2014	Cairo	CS	Older adults home	GDS-15 > 4	240	67.5	Female sex, low social class, insufficient income, partial independence, and loneliness feeling.
El-Bilsha [23]	2018	Dakahlia, Damietta and El-Gharbia	CS	Older adults home	GDS-15 > 4	110	60.0	Socially isolation, loneliness, lack of support system, dissatisfaction with the ageing process and not practicing religious activity regularly.
Mostfa [13]	2019	Cairo	CS	Older adults home	GDS-15 > 4	50	100.0	Decreased daily living activities
El Kady [18]	2013	Alexandria	CS	Geriatric home and hospitalized and community	GDS-15 > 4	100	46.0	Low income and low educational level.
Ahmed [26]	2023	Port Said	CS	Older adults people attending primary health-care centers	GDS-30 > 9	178	49.4	Female sex, high education, unemployment, low income, loneliness, loss of a close person, chronic diseases, tumors, iatrogenic medications, and depression history.
Elnahas [19]	2021	Cairo	CS	Older adults people attending PHC centers	GDS-15 > 4	150	78.0	
Samy [14]	2020	Giza	CS	Older adults people attending PHC centers	GDS-15 > 4	356	71.1	
Elbanouby [25]	2013	Cairo	PC	Hospitalized older adults	GDS-15 > 4	205	50.7	
Esmayel [20]	2018	Zagazig	CS	Hospitalized older adults	GDS-15 > 4	200	72.0	Low income.

*Abbreviations* CS; Cross-sectional, PC; prospective cohort, PHC; Primary health care, GDS-15; Geriatric Depression Scale-15, GDS-30; Geriatric Depression Scale-30

### The prevalence of depression in older adults overall and among different subgroup analyses

**Fig. 2:**
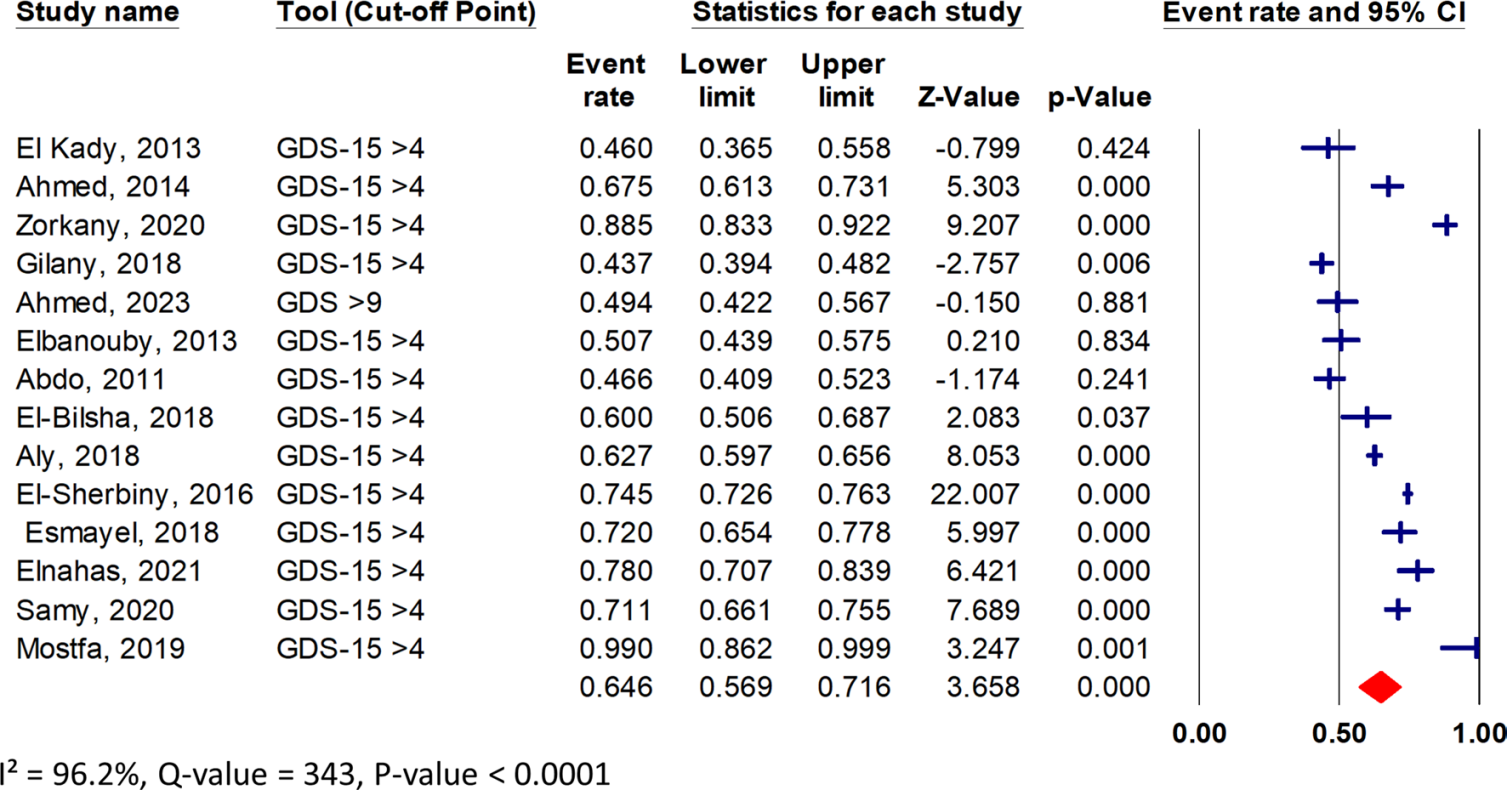
Overall prevalence of depression among Egyptian geriatrics

The pooled prevalence of depression in 14 studies with a total sample size of 5857 was 64.6% (95% CI: 57.0 to 71.5) (Fig. 2). According to the study setting, the older adult population was classified into the following subgroups: Community-dwelling older adults, older adults people attending PHC centers, hospitalized older adults, and older adults residing in geriatric homes. The pooled prevalence of depression was 59.6% (95% CI, 45.6 to 72.2) for community-dwelling older adults, 67.0% (95% CI, 50.0 to 80.4) for older adult people attending PHC centers, 67.0% (95% CI, 48.4 to 81.3) for hospitalized older adults, and 62.0% (95% CI, 44.5 to 76.9) for older adults living in geriatric homes. In terms of marital status, the prevalence of depressive symptoms was highest among divorced individuals at 88.0%, with single, widowed, and married individuals experiencing pooled prevalence of 80.0%, 73.6%, and 55.2%, respectively. The high I-squared value indicated substantial heterogeneity among the included studies, suggesting variations in the prevalence estimations of depression across the overall and subgroup analysis. The forest plots of the subgroup analysis are presented in Figs. S1–S8. See the supplementary file. The prevalence of depressive symptoms overall and subgroups with heterogeneity statistics was presented in Table 2.

**Table 2:** Meta-analysis of the prevalence of depression in overall and study subgroups

Study subgroup	Included studies& citations	Total sample size	Pooled prevalence (%), 95% CI	I squared (%)	Q-value, *P*-value
Overall	14	5857	64.6 (57.0 to 71.5)	96.2	343.0, < 0.001
Based on the study setting					
Community-dwelling older adults	6 [15, 16, 18, 21, 22, 24]	4302	59.6 (45.6 to 72.2)	98.2	281.4, < 0.001
Older adults people attending PHC centers	3 [14, 19, 26]	684	67.0 (50.0 to 80.4)	94.1	34.3, < 0.001
Hospitalized older adults	3 [18, 20, 25]	438	67.0 (48.4 to 81.3)	91.3	22.9, < 0.001
Older adults residing in geriatric homes	4 [17, 18, 23, 26]	433	62.0 (44.5 to 76.9)	84.8	19.9, < 0.001
Based on marital status					
Married individuals	7 [15–18, 21, 24, 26]	1359	55.2 (39.3 to 70.1)	96.6	180.3, < 0.001
Widows	4 [17, 18, 21, 26]	882	73.6 (55.0 to 86.5)	94.2	52.2, < 0.001
Divorced individuals	3 [16–18]	124	88.0 (53.0 to 91.7)	77.6	9.0, 0.011
Single individuals	3 [16, 18, 24]	95	80.0 (63.3 to 90.2)	71.3	7.0, 0.030

*Abbreviations* PHC; Primary health care, CI; Confidence interval

### Risk factors associated with depression among Egyptian geriatrics

For further identification of risk factors for depression in Egyptian geriatrics, we performed a meta-analysis of the risk differences for risk factors reported in at least three studies.

**Fig. 3:**
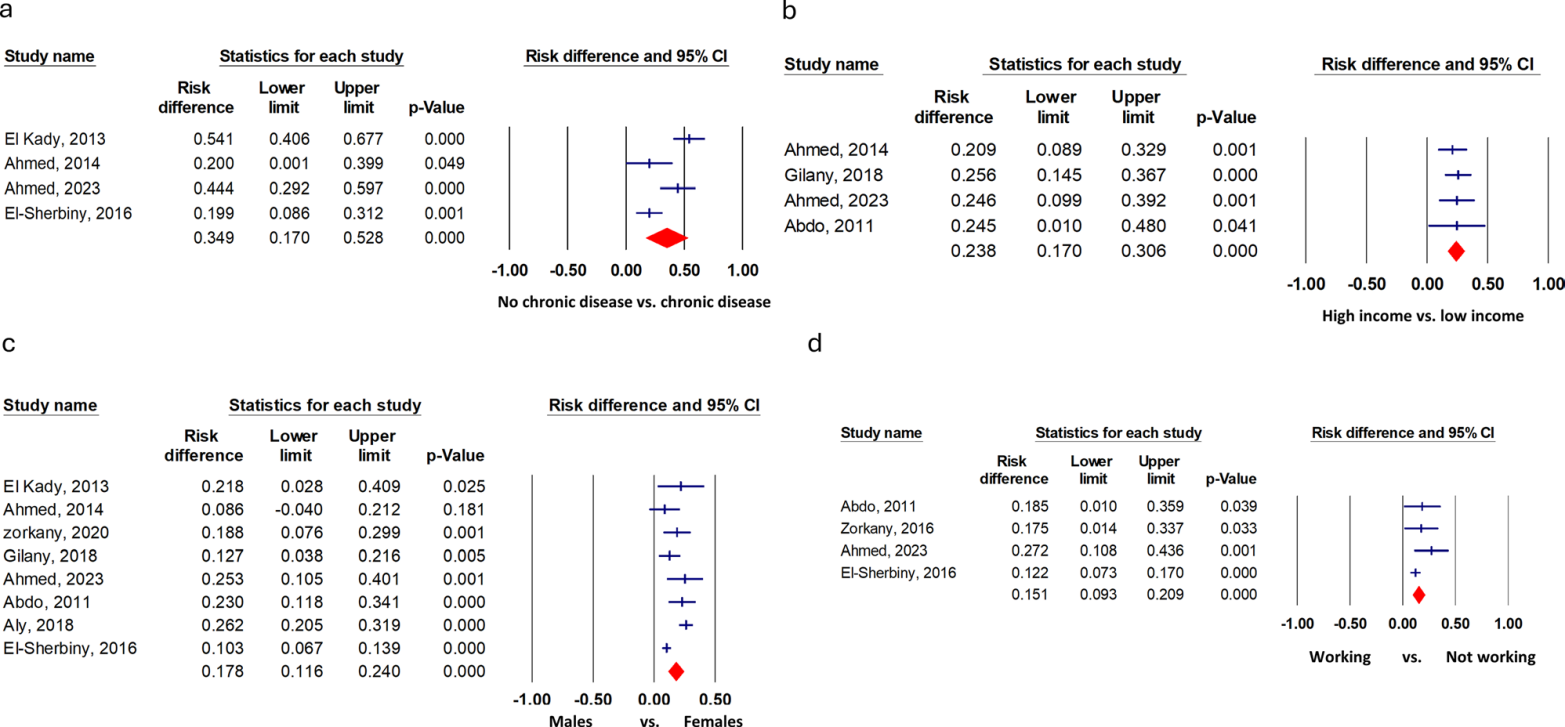
Risk difference for depression in Egyptian geriatrics that showed statistical significance. (**a**) Older adults with chronic illnesses versus those without chronic diseases, (**b**) low-income older adults compared to high-income older adults, (**c**) female older adults compared to male older adults, and (**d**) older adults who are not working versus working older adults.

**Fig. 4:**
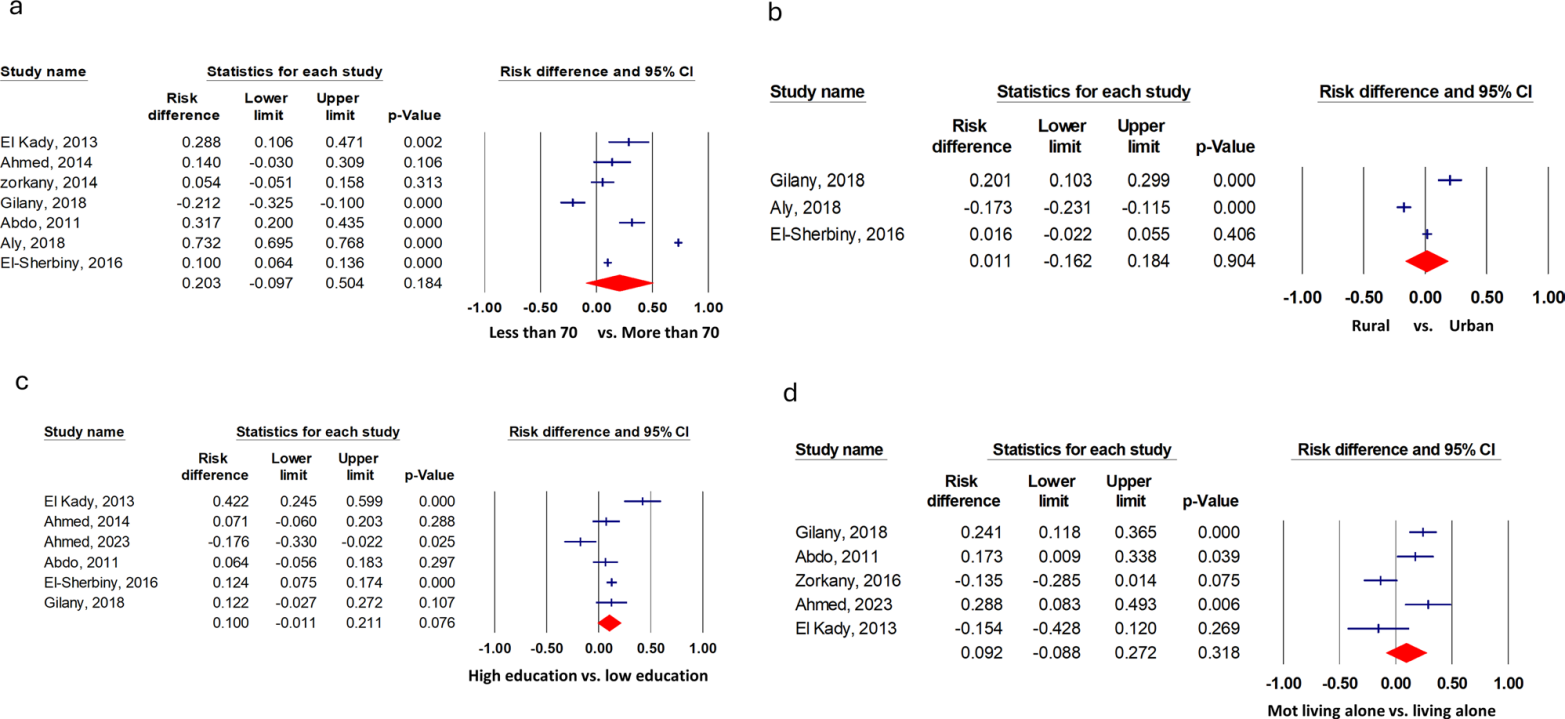
Risk difference for depression in Egyptian geriatrics that showed no statistical significance.(**a**) Older adults aged over 70 compared to those under 70, (**b**) older adults residing in urban areas versus those in rural areas, (**c**) individuals with no formal education or a basic educational background (below secondary school) compared to those with secondary school or university education, and (**d**) older adults living alone versus those who do not.

Older adults patients with chronic illness, female sex, low-income elders, and elders who were unemployed or not working had a higher significant risk for depression compared to elders without chronic disease, male sex, high-income older adults, and those who were working, with pooled risk differences of 34.9% (17.0 to 52.8, 17.8% (11.6 to 24.0), 23.8 (17.0 to 30.6) and 15.1% (9.3 to 20.9) respectively (Fig. 3). However, there was no significant difference in risk for depression in the older adults residing in urban areas compared to rural areas, the older adults aged + 70 compared to those less than 70, individuals with low levels of education or who are illiterate compared with those with higher levels of education and the older adults who live alone compared with those living with family, with a pooled risk difference of 1.1 (-16.2 to 18.3), 20.3 (-9.6 to 50.3), 10.0 (-1.1 to 20.1), and 9.2 (-8.9 to 27.1), respectively (Fig. 4). The pooled risk difference and heterogeneity statistics are provided in Table 3.

**Table 3:** Meta-analysis of risk difference

Risk factor	Included studies	Pooled risk difference (%), 95%CI	I squared (%)	Q-value, *P*-value
**Significant risk factors**				
Chronic diseases vs. absence of chronic diseases	4	34.9 (17.0 to 52.8)	76.1	39.1, < 0.001
Females vs. males	8	17.8 (11.6 to 24.0)	74.1	27.1, < 0.001
Low-income vs. high-income	4	23.8 (17.0 to 30.6)	0.0	0.3, 0.953
Unemployed or not working vs. employed.	4	15.1 (9.3 to 20.9)	14.0	3.5, < 0.001
**Non-Significant risk factors**				
Urban vs. rural	3	1.1 (-16.2 to 18.4)	95.9	49.3, < 0.001
Older adults aged + 70 vs. less than 70	7	20.3 (-9.6 to 50.3)	99.1	737.6, < 0.001
Illiterate or < secondary vs. ≥ secondary	6	10.0 (-1.1 to 20.1)	81.1	26.4 < 0.001
Older adults who live alone vs. not	5	9.2 (-8.8 to 27.1)	81.6	21.8, < 0.001

*Abbreviations* Vs.: Versus, CI; Confidence interval

### Other reported risk factors

Other risk factors were identified for depression among Egyptian geriatrics as formerly mentioned in Table 1. Lack of social support [17, 23], absence of religiosity [15, 23], smoking [16, 21], impairment in activities of daily living [13, 17, 23], insomnia [15, 23], sleeping disturbance [16], social isolation [23] depression history [26], loss of a close person [24, 26], and financial dependence [15, 18].

### Sensitivity analysis

**Fig. 5:**
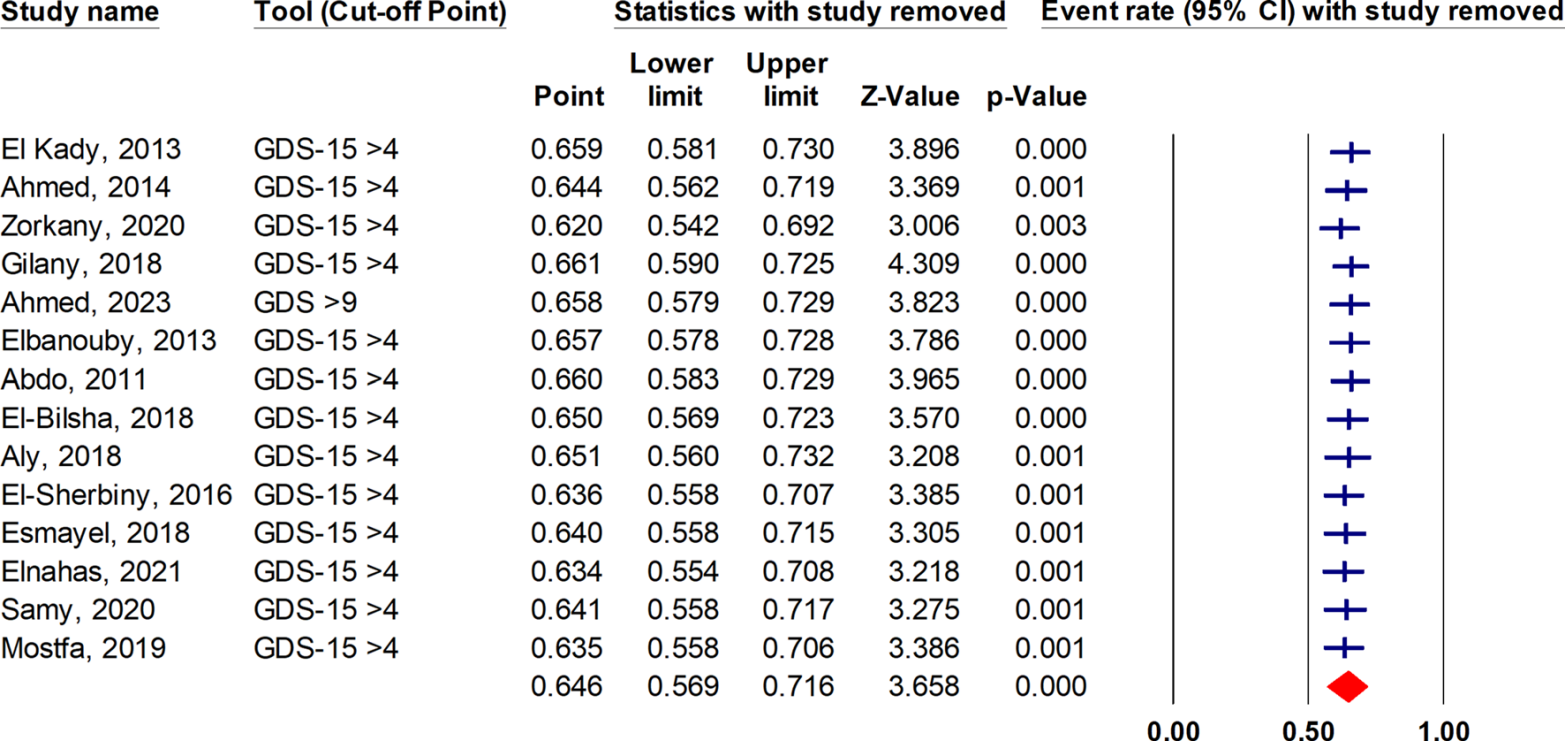
Sensitivity analysis of the overall prevalence of depression in geriatrics

Sensitivity analysis using the leave-one-out approach indicated that the combined estimate of overall depression prevalence is reliable and is not dependent on any one study, as depicted in Fig. 5.

## Discussion

Our study included 14 articles with 5857 older adults and highlighted the following findings: (1) The pooled prevalence of depression among Egyptian geriatrics was 64.6%. (2) The pooled prevalence of depression was 59.6% for community-dwelling older adults, 67.0% for older adults attending PHC centers, 67.0% for hospitalized older adults, and 62.0% for older adults living in geriatric homes. (3) In terms of marital status, the prevalence of depressive symptoms was highest among divorced individuals at 88.0%, with single, widowed, and married individuals experiencing a pooled prevalence of 80.0%, 73.6%, and 55.2%, respectively. (4) Older adult patients with chronic illness, female sex, low-income elders, and elders who were unemployed or not working had a significantly higher risk for depression compared to elders without chronic disease, male sex, high-income older adults, and those who were working, with pooled risk differences of 34.9%, 17.8%, 23.8%, and 15.1%, respectively. (5) There was no significant difference in risk for depression in the older adults residing in urban areas compared to rural areas, the older adults aged + 70 compared to those less than 70, individuals with low levels of education or who are illiterate compared with those with higher levels of education and the older adults who live alone compared with those living with family.

A prior systematic review addressed the prevalence of mental and neurocognitive disorders in Egyptian older adults; however, it was limited by its lack of quantitative synthesis, and the small number of studies included encompassing depression (6 studies) [27]. In the current study, we employed a robust meta-analysis approach encompassing 14 studies, stratifying the prevalence based on the study setting and marital status and thoroughly investigating all the reported risk factors, yielding generalizable findings on prevalence and risk factors. Moreover, our results diverge from the prior review on the association between age, education, and depression. Whereas the earlier study suggested a positive link [27], our meta-analysis analysis with a larger data pool reveals no significant association between these factors and depression.

The GDS is specifically designed for older people and is the most commonly used self-rating scale for geriatric depression in epidemiological studies [28, 29]. It was validated in Arabic with strong psychometric properties [30]. Its ease of administration, combined with proven reliability and sensitivity to the cultural nuances of Arabic-speaking individuals, made the GDS widely adopted for depression screening in Egypt, particularly in healthcare settings where resources were limited [30].

The predictive validity of the GDS as a screening tool for depression in individuals aged 65 years and older was assessed in a recent systematic review and meta-analysis [31]. The study revealed that the sensitivity and specificity of the GDS-15 were 0.80 (95% CI: 0.7 to 0.8) and 0.79 (95% CI: 0.78 to 0.80), respectively (with cut-off scores varying between 3 and 7). These results indicated that the GDS-15 continues to exhibit a diagnostic value for older adults, but diagnostic accuracy is slightly lower among older adults with cognitive impairment [31]. Notably, all the included articles in our study assessed depression in geriatrics using the shorter version of the GDS (GDS-15), with a cut-off score of more than 4, suggesting depression, except for one study that used the longer version of the GDS (GDS-30), with a cut-off score of more than 9, suggesting clinically relevant depression [26].

When compared to other studies, the combined prevalence of depression among Egyptian geriatric individuals (64.6%) was higher than the pooled prevalence observed in similar meta-analyses conducted in Iran, India, and China, which reported GDS-based prevalence estimations of 48.0%, 37.9%, and 27.3%, respectively [6–8]. These differences may be due to variations in socio-cultural or economic factors, health infrastructure, awareness, or even methodological differences.

According to the current study, depression was higher in older adult patients with chronic illness than in patients without chronic disease, with a pooled risk difference of 34.9%. There is a bidirectional relationship between depression and chronic illnesses [32]. The presence of this comorbidity is correlated with an increased risk of mortality and reduces the effectiveness of therapies [32]. Chronic diseases can both cause and worsen depression due to the burdens associated with the condition that significantly impacts the affected individuals’ quality of life [33–36]. In turn, depression amplifies the severity of the chronic illness by impeding self-care practices and adherence to medical treatments [33–36]. Another important risk factor in the current study was low socioeconomic conditions, which play an important role in influencing the mental health of older adults in low- and middle-income countries [37]. Additionally, females exhibit a higher risk for depressive symptoms than males. This may be attributed to hormonal changes that make women more vulnerable to depression (postmenopausal depression) [38]. Moreover, factors such as financial dependence, widowhood, and caregiving responsibilities tend to be more prevalent among females, further contributing to their increased risk of developing depression [39].

On the other hand, there was no significant difference in risk for depression between the older adults residing in urban areas compared to rural areas. Among the three included studies, one revealed that older adults living in urban areas had a higher risk for depression [15]. The opposite was reported by another study, where rural residence was a risk factor for depression [22], while a non-significant result was obtained by the third study, where the prevalence was almost the same in urban and rural areas [21]. Similarly, there is no significant difference in risk between individuals over 70 years old and those below 70 years old. Though the majority of the included studies in our analysis reported increased age as a risk factor for depression, these studies did not adjust for confounding factors, in particular chronic diseases. Therefore, we suppose that rather than ageing, what seems to be age-related impacts on depression might be attributable to the rise in physical health issues and associated disabilities and or socioeconomic status. Individuals with lower levels of education or who are unable to read and write, exhibited a non-significant combined risk difference in comparison to those with higher levels of education. Among the six studies included, two of them consistently identified low education as a significant risk factor for depression [18, 21]. Non-significant association in three studies [15, 17, 24]. One study, however, found the contrary to be true, suggesting that higher education is, in fact, a risk factor [26]. In addition, there was no significant difference in risk for the older adults who live alone compared with those living with family. Among the five studies examined, two of them indicated that older adults living alone exhibit lower levels of depression compared to those living with their families [16, 18]. In contrast, three studies reported contrasting findings, suggesting that older adults living with their families experience lower levels of depression than those living alone [15, 24, 26]. These conflicting results underscore the need for additional research to establish a solid understanding of the relationship between these factors and depression among Egyptians.

### Limitations

It is critical to recognize some constraints. First, there is a scarcity of studies focusing on Upper Egypt, the country’s southern region, which is characterized by limited access to healthcare, deeply rooted traditional norms, higher illiteracy rates, and greater levels of poverty compared to Lower Egypt [40]. This expected underrepresentation may result in an underestimation of the pooled prevalence of depressive symptoms among older adults in Egypt. Second, there was inter-study heterogeneity, which is unavoidable in epidemiological meta-analyses. Finally, while screening approaches can benefit public health efforts, they cannot substitute a complete clinical interview for confirming a depression diagnosis.

### Recommendations for future research

Future studies should consider utilizing diagnostic tools, such as the Diagnostic and Statistical Manual of Mental Disorders (DSM) or the International Classification of Diseases (ICD), to provide a more comprehensive and precise assessment of depression in geriatric populations. Furthermore, considering the underrepresentation of Upper Egypt and its distinctive socio-cultural and economic factors in this meta-analysis, it is strongly recommended that future research prioritize this region to achieve a more comprehensive understanding of its mental health landscape. Additionally, researchers should aim to control potential confounders more rigorously through matching or multivariate analyses to enhance the validity of their findings. Longitudinal study designs should be considered to investigate the progression of depression over time and determine the impact of depression on quality of life, healthcare utilization, and morbidity and mortality.

Given the high prevalence of depression and the anticipated increase in the proportion of older Egyptian individuals in the future [10], it is crucial to prioritize the mental health of older adults. We believe that the following factors can help reduce the prevalence of depression among Egyptian geriatrics: First, improving access to mental healthcare, along with public awareness and education. Second, strengthening social support networks for older adults can contribute to better mental health outcomes. Third, implementing initiatives for economic empowerment, such as alleviating poverty, ensuring financial security, and enhancing economic opportunities for older adults, can help reduce the burden of economic stressors that contribute to depression. Lastly, integrating mental health services into PHC, hospitals, geriatric homes, and community-based settings can improve the early detection and management of depression across all care environments.

## Conclusion

In Egypt, more than half of the older adult population exhibits symptoms of depression. The primary risk factors for depression among Egypt’s older adults are chronic illnesses, female sex, unemployment, and low levels of income. Integrating mental health services into PHC, hospitals, geriatric homes, and community-based settings can improve early detection and management of depression due to the detrimental effects it has on health outcomes and overall well-being. In addition, boosting understanding of depression in older adults requires public awareness and education.

## Electronic supplementary material

Below is the link to the electronic supplementary material.

Supplementary Material 1

## Data Availability

No datasets were generated or analysed during the current study.

## Abbreviations

CS: Cross-sectional
PC: Prospective cohort
DSM: Statistical Manual of Mental Disorders
GDS: Geriatric Depression Scale
GDS-15: Geriatric Depression Scale-15
ICD-10: International Classification of Diseases-10
PHC: Primary health care
