# Supplementary material for: Depression in geriatrics: a systematic review and meta-analysis of prevalence and risk factors in Egypt
Source: BMC Geriatr. 2024 Nov 11;24:930. doi: 10.1186/s12877-024-05484-2 (PMC11552400; doi:10.1186/s12877-024-05484-2)
Supplement: Supplementary file 1 — Supplementary Material 1 [file 12877_2024_5484_MOESM1_ESM.docx]

**Supplementary file**

**Depression in geriatrics: a systematic review and meta-analysis of prevalence and risk factors in Egypt**

Hebatalla Ahmed^1*^, Heba Khaled^2^, Hassan Samy Hassan^3^, Bassima Alodini^4^, Ahmed Azzam^5^, Anees adel Hjazeen^6^, Ahmed A. Allam^7^, Sarah Mohamed Hussein^8^, Fatma E. Hassan^9,10^.

^1^ Department of Public Health, Faculty of Medicine, Kafrelshiekh University, Kafrelshiekh, Egypt.
^2^ Department of Biochemistry, Faculty of pharmacy, Cairo University, Cairo, Egypt.
^3^ Faculty of Pharmacy, Tanta University, Tanta, Egypt.
^4^ Biomedicine, Uppsala University, Uppsala, Sweden.
^5^ Department of Microbiology and Immunology, Faculty of Pharmacy, Helwan University, Ain Helwan, Cairo, Egypt
^6^ Biostatistician at Royal Medical Services, Amman, Jordan.
^7^ Department of Biology, Biotechnology Program, the American University in Cairo, New Cairo, Egypt.
^8^ Department of Public Health, Community Medicine, Environmental and Occupational Medicine, Faculty of Medicine, Suez Canal University, Ismailia, Egypt. ^9^ Medical Physiology Department, Kasr Alainy, Faculty of Medicine, Cairo University, Giza 11562, Egypt.

^10^ General Medicine Practice Program, Department of Physiology, Batterjee Medical College, Jeddah 21442, Saudi Arabia

*Corresponding author: Hebatalla Ahmed

Department of Public Health, Faculty of Medicine, Kafrelshiekh University, Kafrelshiekh, Egypt.

Email: [Heba_Abdelmasoud@med.kfs.edu.eg](mailto:Heba_Abdelmasoud@med.kfs.edu.eg)

**Table S1:** Supplementary preferred reporting items for systematic reviews and meta-analyses (PRISMA) checklist.

| Section/topic | Item No | Checklist item | Reported on page No |
| --- | --- | --- | --- |
| Title | 1 | Identify the report as a systematic review, meta-analysis, or both | 1 |
| Abstract | | | |
| Structured summary | 2 | Provide a structured summary including, if applicable, background, objectives, data sources, study eligibility criteria, participants, interventions, study appraisal and synthesis methods, results, limitations, conclusions and implications of key findings, systematic review registration number | 2,3 |
| Introduction | | | |
| Rationale | 3 | Describe the rationale for the review in the context of what is already known | 4 |
| Objectives | 4 | Provide an explicit statement of questions being addressed with reference to participants, interventions, comparisons, outcomes, and study design (PICOS) | 4 |
| Methods | | | |
| Protocol and registration | 5 | Indicate if a review protocol exists, if and where it can be accessed (such as web address), and, if available, provide registration information including registration number | - |
| Eligibility criteria | 6 | Specify study characteristics (such as PICOS, length of follow-up) and report characteristics (such as years considered, language, publication status) used as criteria for eligibility, giving rationale | 5 |
| Information sources | 7 | Describe all information sources (such as databases with dates of coverage, contact with study authors to identify additional studies) in the search and date last searched | 4, 5 |
| Search | 8 | Present a full electronic search strategy for at least one database, including any limits used, such that it could be repeated | 4, 5 |
| Study selection | 9 | State the process for selecting studies (that is, screening, eligibility, included in the systematic review, and, if applicable, included in the meta-analysis) | 4, 5 |
| Data collection process | 10 | Describe the method of data extraction from reports (such as piloted forms, independently, in duplicate) and any processes for obtaining and confirming data from investigators | 5 |
| Data items | 11 | List and define all variables for which data were sought (such as PICOS, and funding sources) and any assumptions and simplifications made | 5 |
| Risk of bias in individual studies | 12 | Describe methods used for assessing the risk of bias in individual studies (including specification of whether this was done at the study or outcome level), and how this information is to be used in any data synthesis | 5  **Tables S2** |
| Summary measures | 13 | State the principal summary measures (such as risk ratio, and difference in means). | 6 |
| Synthesis of results | 14 | Describe the methods of handling data and combining results of studies, if done, including measures of consistency (such as I^2^ statistic) for each meta-analysis | 6 |
| Risk of bias across studies | 15 | Specify any assessment of risk of bias that may affect the cumulative evidence (such as publication bias, selective reporting within studies) | - |
| Additional analyses | 16 | Describe methods of additional analyses (such as sensitivity or subgroup analyses, meta-regression), if done, indicating which were pre-specified | 6 |
| Results | | | |
| Study selection | 17 | Give numbers of studies screened, assessed for eligibility, and included in the review, with reasons for exclusions at each stage, ideally with a flow diagram | 6  **Fig. 1** |
| Study characteristics | 18 | For each study, present characteristics for which data were extracted (such as study size, PICOS, follow-up period) and provide the citations | 7  **Table 1** |
| Risk of bias within studies | 19 | Present data on the risk of bias of each study and, if available, any outcome-level assessment (see item 12). | **Table S2** |
| Results of individual studies | 20 | For all outcomes considered (benefits or harms), present for each study (a) simple summary data for each intervention group and (b) effect estimates and confidence intervals, ideally with a forest plot | 8  Figs. 2-4 and Tables 2&3 |
| Synthesis of results | 21 | Present results of each meta-analysis done, including confidence intervals and measures of consistency | 8  Figs. 2-4 and Tables 2&3 |
| Risk of bias across studies | 22 | Present results of any assessment of risk of bias across studies (see item 15) | - |
| Additional analysis | 23 | Give results of additional analyses, if done (such as sensitivity or subgroup analyses, meta-regression) (see item 16) | 8, Fig. 5 |
| Discussion | | | |
| Summary of evidence | 24 | Summarize the main findings including the strength of evidence for each main outcome; consider their relevance to key groups (such as health care providers, users, and policymakers) | 10-11 |
| Limitations | 25 | Discuss limitations at the study and outcome level (such as the risk of bias), and at the review level (such as incomplete retrieval of identified research, reporting bias) | 13 |
| Conclusions | 26 | Provide a general interpretation of the results in the context of other evidence, and implications for future research | 14 |
| Funding | | | |
| Funding | 27 | Describe sources of funding for the systematic review and other support (such as the supply of data) and the role of funders for the systematic review | 15 |

**Table S2:** The checklist items for Joanna Briggs's critical appraisal tool for prevalence studies.

|  | Yes | No | Unclear | Not applicable |
| --- | --- | --- | --- | --- |
| 1. Was the sample frame appropriate to address the target population? | □ | □ | □ | □ |
| 1. Were study participants sampled in an appropriate way? | □ | □ | □ | □ |
| 1. Was the sample size adequate? | □ | □ | □ | □ |
| 1. Were the study subjects and the setting described in detail? | □ | □ | □ | □ |
| 1. Was the data analysis conducted with sufficient coverage of the identified sample? | □ | □ | □ | □ |
| 1. Were valid methods used for the identification of the condition? | □ | □ | □ | □ |
| 1. Was the condition measured in a standard, reliable way for all participants? | □ | □ | □ | □ |
| 1. Was there appropriate statistical analysis? | □ | □ | □ | □ |
| 1. Was the response rate adequate, and if not, was the low response rate managed appropriately? | □ | □ | □ | □ |

**Table S3:** Quality assessment of the included studies.

| **Author** | **Checklist item** | | | | | | | | | **Score**  **(Out of 9)** |
| --- | --- | --- | --- | --- | --- | --- | --- | --- | --- | --- |
|  | **1** | **2** | **3** | **4** | **5** | **6** | **7** | **8** | **9** |  |
| Zorkany[14] (2020) | 1 | 1 | 0 | 1 | 1 | 1 | 1 | 1 | 1 | 8 |
| Gilany[13] (2018) | 1 | 1 | 1 | 1 | 1 | 1 | 1 | 1 | 1 | 9 |
| Abdo[22] (2011) | 1 | 0 | 0 | 1 | 1 | 1 | 1 | 1 | 1 | 7 |
| Aly[20] (2018) | 1 | 1 | 1 | 1 | 1 | 1 | 1 | 1 | 1 | 9 |
| El-Sherbiny[19] (2016) | 1 | 1 | 1 | 1 | 1 | 1 | 1 | 1 | 1 | 9 |
| Ahmed[15] (2014) | 1 | 1 | 0 | 1 | 1 | 1 | 1 | 1 | 1 | 8 |
| El-Bilsha[21] (2018) | 1 | 0 | 0 | 1 | 1 | 1 | 1 | 1 | 1 | 7 |
| Mostfa[11] (2019) | 1 | 0 | 0 | 0 | 1 | 1 | 1 | 1 | 1 | 6 |
| El Kady[16] (2013) | 1 | 1 | 0 | 1 | 1 | 1 | 1 | 1 | 1 | 8 |
| Ahmed[24] (2023) | 1 | 1 | 0 | 1 | 1 | 1 | 1 | 1 | 1 | 8 |
| Elnahas[17] (2021) | 1 | 0 | 0 | 1 | 1 | 1 | 1 | 1 | 1 | 7 |
| Samy[12] (2020) | 1 | 0 | 1 | 1 | 1 | 1 | 1 | 1 | 1 | 8 |
| Elbanouby[23] (2013) | 1 | 0 | 0 | 1 | 1 | 1 | 1 | 1 | 1 | 7 |
| Esmayel[18] (2018) | 1 | 0 | 0 | 1 | 1 | 1 | 1 | 1 | 1 | 7 |

**Fig. S1**


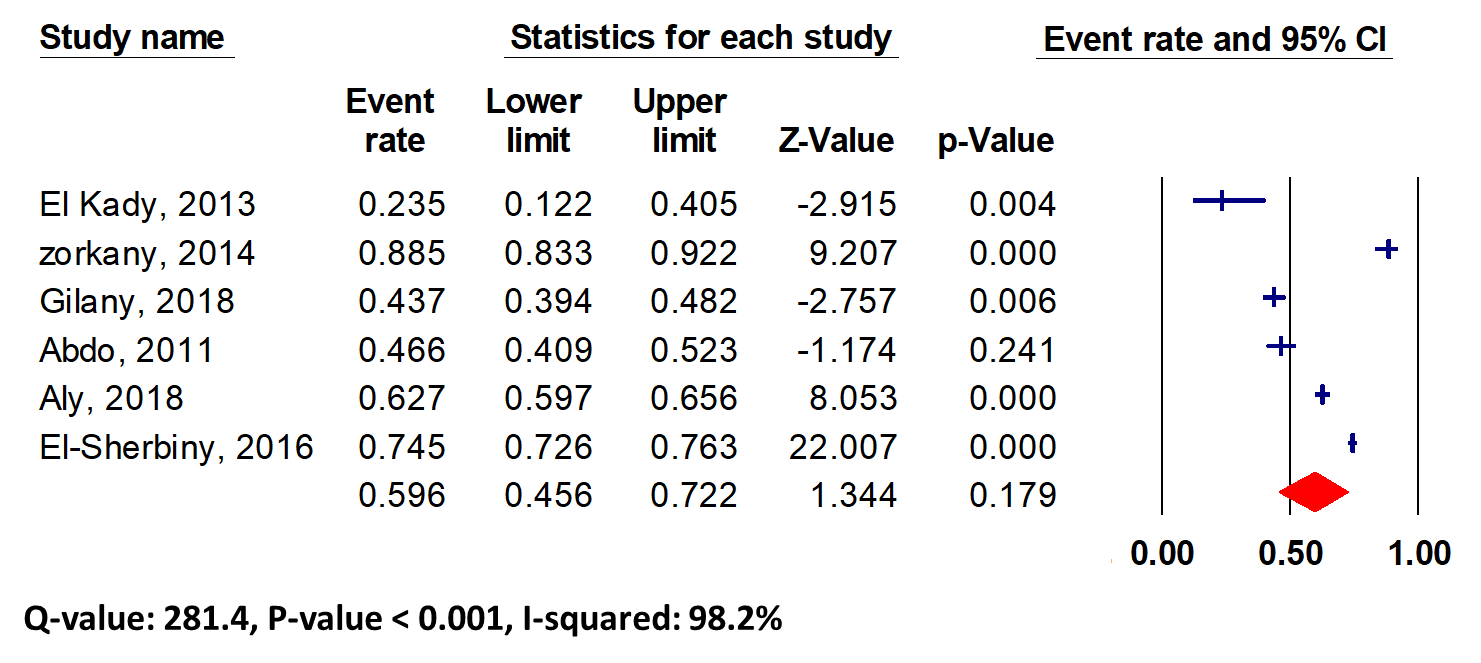


**The prevalence of depressive symptoms among community dwelling elderly**

**Fig. S2**


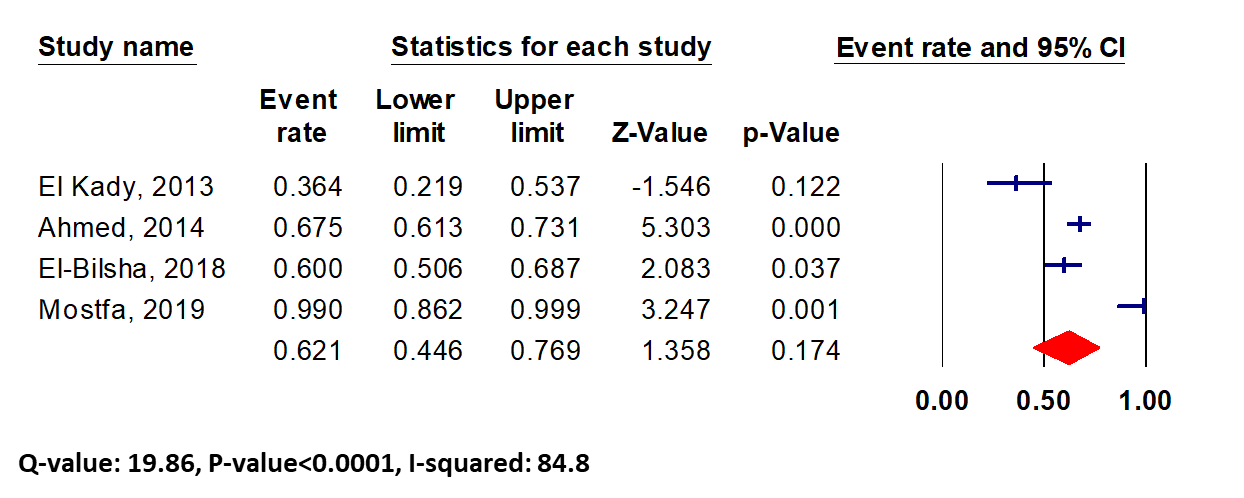


**The prevalence of depressive symptoms among elderly residing in geriatric home**

**Fig. S3**


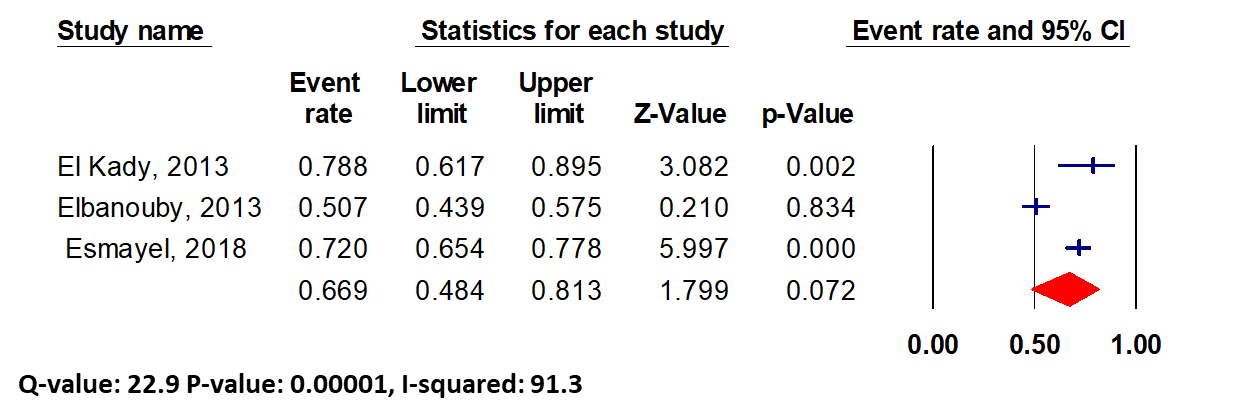


**The prevalence of depressive symptoms among hospitalized elderly**

**Fig. S4**


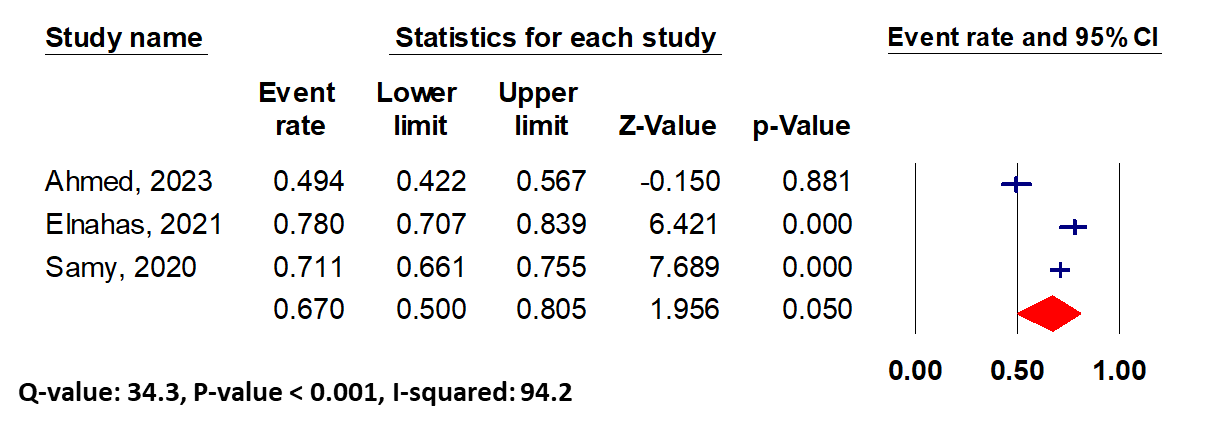


**The prevalence of depressive symptoms among Elderly people attending Primary health care centers**

**Fig. S5**


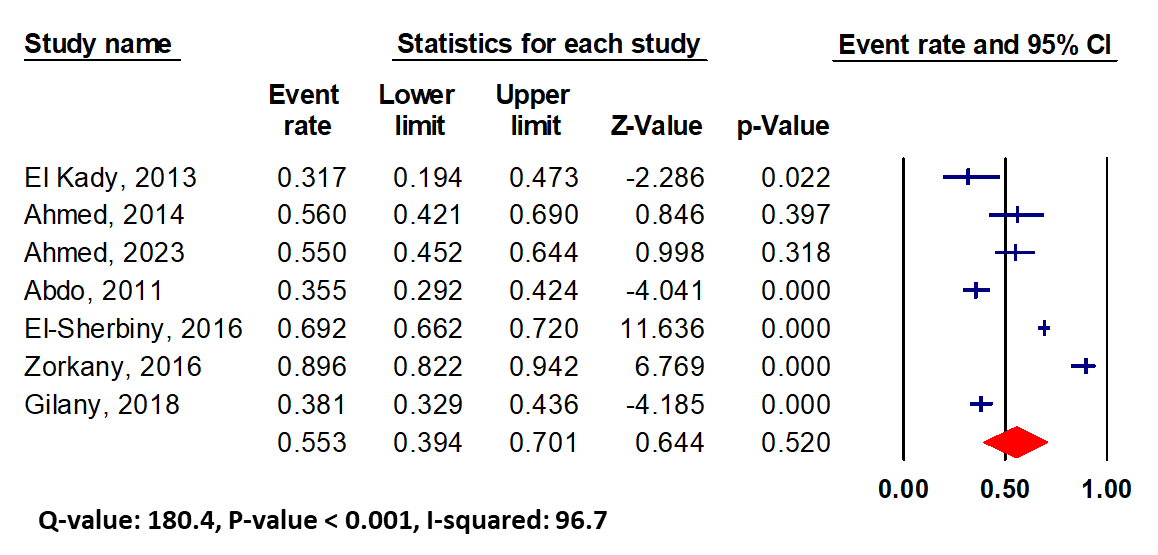


**The prevalence of depressive symptoms among married elderly**

**Fig. S6**


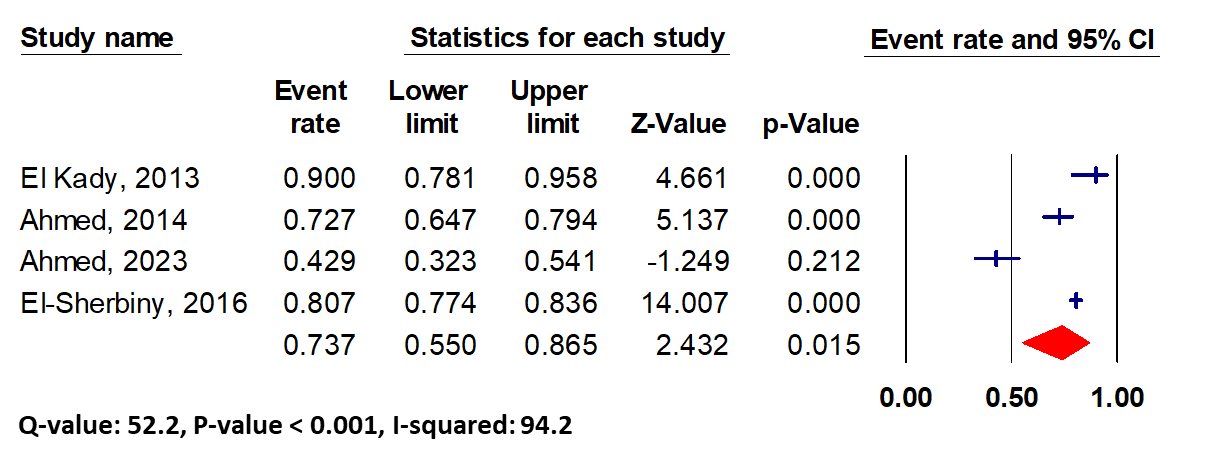


**The prevalence of depressive symptoms among widows of the elderly**

**Fig. S7**


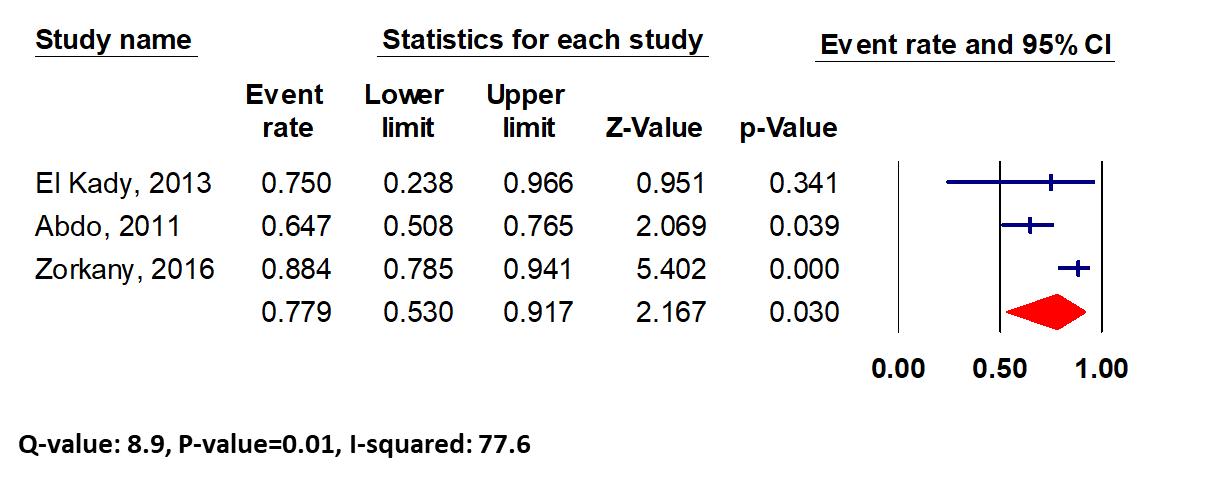


**The prevalence of depressive symptoms among divorced elderly**

**Fig. S8**


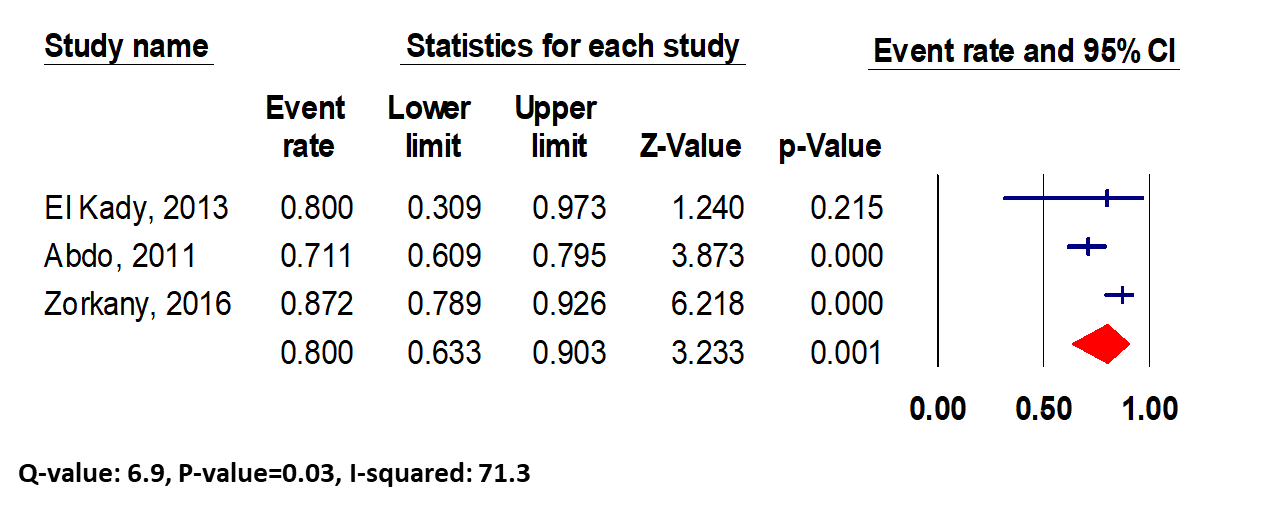


**The prevalence of depressive symptoms among single elderly**
